# The Irish Anti-College Scheme

**Published:** 1917-05-26

**Authors:** 


### THE EDITOR'S LETTER-BOX.

### THE IRISH ANTI-COLLEGE SCHEME.

*To the Editor of* The Hospital.

Sir,—Your readers will be interested to know that the Irish "antis" have shot their bolt. Having remained somnolent all these years, the trumpet-note of the Royal British College of Nursing aroused them to action; and, behold! they have launched a scheme—a scheme backed by the College of Surgeons but rejected by the College of Physicians. It is an extremely simple and innocuous affair, and contains only one blot, but this, I am afraid, will prove fatal to its success.

The scheme consists of forming an "Irish Nursing Board," and its objects are stated to be as follows :—

1. To promote the education of Irish nurses.
2. To examine such persons as shall have received an education that this Board shall consider sufficient.
3. To grant certificates to such persons as shall have passed the prescribed examination of the Board.
4. To make and to maintain a Register of such persons as shall have received the certificate of the Board.
5. To remove from the Register, if thought fit, the name of any person who shall be convicted in England or Ireland of any felony or misdemeanour, or in Scotland of any crime or offence, or who shall be judged by the Board to have been guilty of grave misconduct in any professional respect.

There are published in their circular announcing this the names of 119 nurses, largely matrons and private nurses, and from these twenty-two persons are to be elected, who, with four persons to be elected by the Royal College of Surgeons, will form the first Board. The 119 names carry no weight or significance, inasmuch as they do not indicate support or approval of the scheme. As a matter of fact, they include the names of all those ladies who belong to the Irish Board of the Royal British College of Nursing. This first Board, when elected, is to continue in office for one year after the opening of the Register, after which the twenty-two nursing members are to be "elected by ballot by the persons whose names are on the Register of the Board."

The Fatal Blot.

If this scheme were free, many Irish nurses might give it support. It would enable them to enrol on a Register of some sort, and sit on the fence watching the development and proceedings of the Royal British College of Nursing. But there is an entrance fee of a guinea, and I am afraid very few Irish nurses will see the fun of paying this sum in order to exchange their diplomas for new ones. I refer, of course, to trained nurses, a remark which I make advisedly, inasmuch as the clause announcing the guinea fee is so worded that anybody and everybody who chooses to pay a guinea may find a welcome. "Up to May 31, 1918," runs the clause, "all persons who can satisfy the Board of their competence as nurses, and who are otherwise proper persons, shall have their names placed on the Register on payment of a fee of one guinea." The omission here of all reference to training is very remarkable, and has all the appearance of being deliberate and designed so as to attract guineas rather than women with high qualifications. The next clause accentuates this view. "From May 31, 1918, to May 31, 1919, all persons who shall have completed their training before the end of one year after the opening of the Register, who can satisfy the Board of their competence as nurses, and who are otherwise proper persons, shall have their names placed on the Register on the payment of a fee of four guineas." In other words, nurses eligible for registration, but who fail to register during the first year, are threatened with a four-guinea fee.

The Alternatives.

It comes to this, that any Irish nurse who wishes to register either with the Irish College of Surgeons or with the Royal British College of Nursing will have to pay a guinea. One is purely local—parochial, in fact. The other will link its members with the whole of the British Empire. The Irish scheme is initiated by a coterie who have never lifted a finger for the benefit of the profession save and except to profess their adherence to the principle of State Registration. In these days of stress and difficulty, when brothers and mothers stretch their hands across the seas to show their comradeship, and that, despite the distances, there is still a union of hearts—in such times the policy of voluntary isolation appears to be singularly out of place. One is reminded of Pope's mouse—

"The mouse that always trusts to one poor hole
Can never be a mouse of any soul."

If I may venture to make a prophecy it is that very few of the younger generation of nurses will regard the investment of a guinea in the Irish Board as a profitable transaction. They would receive in return a guinea's worth of Nothing. IERNE.

### Warrior Patients in Hospitals: their Temperatures and Maladies.

*To the Editor of* The Hospital.

